# Two novel *ECHS1* variants, affecting splicing and reducing enzyme activity, is associated with mitochondrial encephalopathy in infant: a case report

**DOI:** 10.1186/s12883-020-01735-y

**Published:** 2020-04-30

**Authors:** Miaojuan Wu, Wenqi Gao, Zhifang Deng, Zhisheng Liu, Jiehui Ma, Han Xiao, Yu Xu, Dan Sun

**Affiliations:** 1 Jianghan University, Wuhan, Hubei China; 2 Institute of Maternal and Child Health, Wuhan Children’s Hospital (Wuhan Maternal and Child Healthcare Hospital), Tongji Medical College, Huazhong University&Technology, Wuhan, Hubei China; 3 Department of Pharmacy, The Central Hospital of Wuhan, Tongji Medical College, Huazhong University of Science&Technology, Wuhan, Hubei China; 4 Department of Pediatric Neurology, Wuhan Children’s Hospital (Wuhan Maternal and Child Healthcare Hospital), Tongji Medical College, Huazhong University&Technology, Wuhan, Hubei China; 5 Department of Nosocomial Infection, Wuhan Children’s Hospital, Tongji Medical College, Huazhong University&Technology, Wuhan, Hubei China

**Keywords:** Mitochondrial encephalopathy, ECHS1, Variants

## Abstract

**Background:**

Short-chain enoyl-CoA hydratase (ECHS1) is a multifunctional mitochondrial matrix enzyme involved in the second step of mitochondrial fatty acid β-oxidation. Mitochondrial diseases resulting from *ECHS1* mutations are often characterised by encephalopathy, deafness, epilepsy, optic atrophy, cardiomyopathy, dystonia, and lactic acidosis. In this study, we report two novel heterogeneous variants, c.414 + 5G > A (in intron 3) and c.310C > G (in CDS), of *ECHS1* in an infant with mitochondrial encephalopathy.

**Case presentation:**

The two novel variants, c.414 + 5G > A (Chr10:135183403) in intron 3 and c.310C > G (Chr10:135183512) in CDS, were identified by next generation sequencing (NGS). A minigene assay was used to analyse the function of the c.414 + 5G > A variant. ECHS1 enzyme activity was measured by spectrophotometry in the patient-derived myoblasts. The 2-year old patient presented with mitochondrial encephalopathy since birth. Clinical features were encephalopathy, epilepsy, and hindered psychomotor and language development. Serum lactate and blood ammonia levels were elevated, and brain magnetic resonance imaging showed abnormal signals in the bilateral frontal, parietal, and occipital cortices and brainstem and basal ganglia. We found two novel heterogeneous variants in *ECHS1* in this patient. Minigene assay revealed the c.414 + 5G > A variant as the cause of intronic cryptic splice site activation and 39 bp deletion in mature mRNA. In silico analysis predicted that c.310C > G might change glutamine (Q) to glutamic acid (E) in the 104th amino acid sequence (p.Q104E). To investigate the impact of these two variants on protein function, we constructed a 3D model of human ECHS1 and showed that the variants might alter the highly conserved region in close proximity to the active site, which might hinder, or even halt, enzymatic activity. The experimental assay showed that ECHS1 enzyme activity in the patient-derived myoblasts decreased compared to that in control.

**Conclusions:**

Our findings are the first to report a mitochondrial encephalopathy infant carrying two novel *ECHS1* variants, c.414 + 5G > A and c.310C > G, which might be deleterious variants, function as pathogenicity markers for mitochondrial encephalopathy, and facilitate disease diagnosis.

## Background

Mitochondrial disorders, mostly genetically heterogeneous, include different clinical phenotypes. Brain involvement is commonly found in most cases, but rarely is the unique clinical manifestation. Therefore, it is difficult to make a definitive diagnosis of mitochondrial disorders in patients, especially infants. Various genetic defects in nuclear genes that encode mitochondrial proteins could cause mitochondrial dysfunction, ultimately resulting in mitochondrial diseases. It is important for medical staff to know the association between clinical manifestations and genetic testing of mitochondrial disorders to establish accurate diagnoses.

*ECHS1*, a nuclear gene, is located on chromosome 10q26.2-q26.3 [1, 2]. The *ECHS1* gene encodes the mitochondrial short-chain acryloxyethyl CoA hydratase (short-chain enoyl-CoA hydratase, SCEH, or ECHS1), which is localised in the mitochondrial matrix and catalyses the hydration of enoyl-CoA in many metabolic pathways, including short-chain fatty acid β-oxidation, branched-chain amino acid catabolism, and mitochondrial enzyme catalytic unsaturated fatty acids [3, 4]. *ECHS1* has been extensively investigated in model organisms due to its special role in mitochondrial oxidation; however, the relationship between *ECHS1* and infant health and disease is obscure.

In this study, we reported two novel *ECHS1* variants and provided experimental evidence that attests to the functional significance of these variants and their association with severe encephalopathy.

## Case presentation

A 2-year old patient was hospitalised in Wuhan Children’s Hospital (Wuhan Maternal and Child Healthcare Hospital). Signatures of written informed consent forms by the patients and their family members were acquired, and the study was approved by the review boards of the ethics institutions.

The patient reported was an infant boy with healthy and non-related parents, born after a 40-week pregnancy (weight 3500 g, length 51 cm). This patient was the first affected child in his family (his parents had two children, his elder brother was unaffected). His parents were both Chinese and not cousins. When he was born, a hug-like disease attack began, occurring 5–6 times a day, frequently at night. Subsequently, at 1 month of age, the patient was hospitalised due to neonatal pneumonia, neonatal jaundice, and left hydronephrosis. Psychomotor developmental delay was noted at 8 months of age. He could not raise his head at 8 months of age, and could not sit alone at 13 months of age, or speak a meaningful word when he was 2 years old, with muscle hypotonia and spasticity becoming prominent after the first year.

Electromyography (EMG) and repeated EEG recordings were normal. Laboratory tests showed elevated lactic acid- 11.41 mmol/L (normal range 0.5–2.22 mmol/L)- and blood ammonia- 201 μmol/L (normal range 18–72 μmol/L). Blood acylcarnitine analysis showed no abnormality. The value of blood total ketone body of this infant is 0.2 mmol/L. Urinary organic acid profiling revealed elevated 3-hydroxybutyrylcarnitine excretion. Urine analysis showed KET^3+^, BIL^1+^, and PRO^1+^. A positive result in the fecal occult blood test suggested that intestinal mucosal damage was present. Myocardial enzyme, electrolyte, T3, T4, and TSH levels were within the normal range, and liver and renal functions were basically normal. Our diagnosis was “mitochondrial myopathy”, and we administered the appropriate therapeutic cocktail for about 3 months. After the treatment, there was significant improvement in the symptoms, especially in the hug-like episode frequency.

**Fig. 1:**
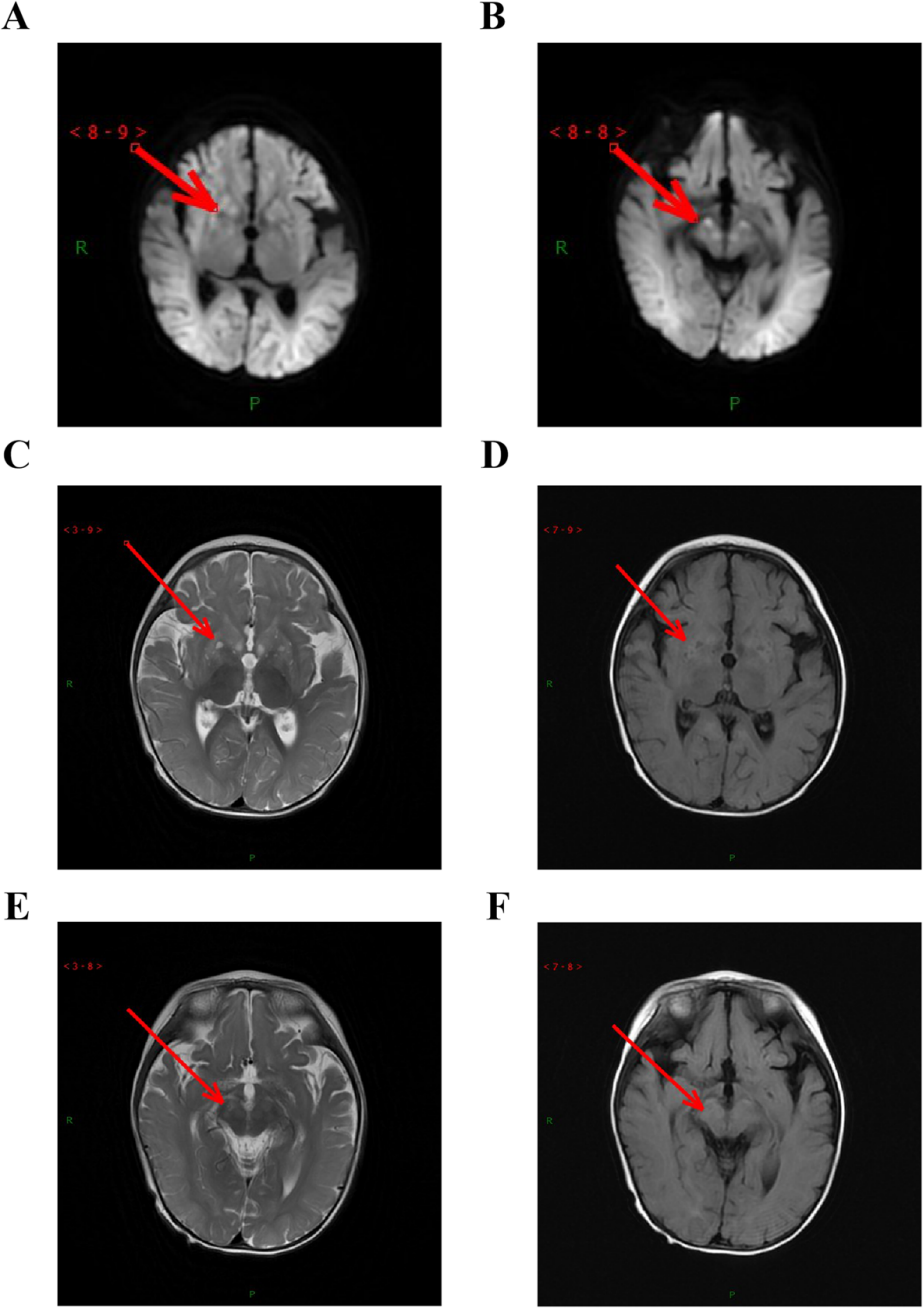
The MRI assay of the patient. (**a**-**b**) DWI image; (**c**-**d**) T2 image;(**e**-**f**)T2 FLAIR image

**Fig. 2:**
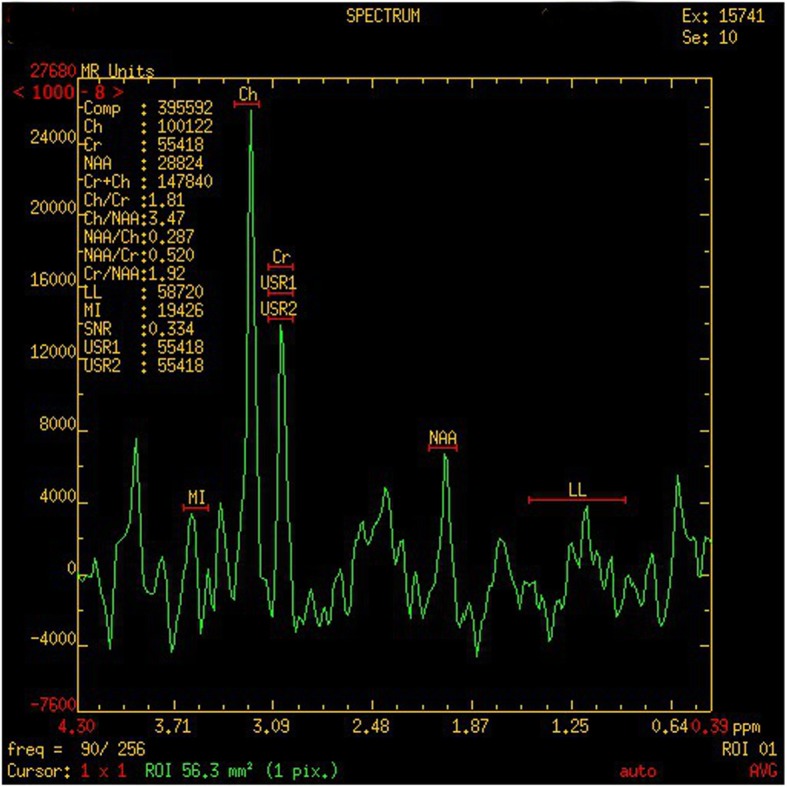
The MRS assay of the patient

Brain magnetic resonance imaging showed diffuse, long T1 and T2 signals distributed symmetrically in the cortex, brainstem, and basal ganglia of the double forehead occipital lobe. The lateral ventricles were slightly enlarged, and the cerebral sulcus was slightly wider. There was no obvious abnormal signal in the cerebellum of the posterior fossa and no shift in the midline structure (Fig. 1). The MRS results indicated a decrease in NAA with a Ch/NAA of 3.47 (Fig. 2).

### Functional analysis of c.414 + 5G > A and c.310C > G variants

**Fig. 3:**
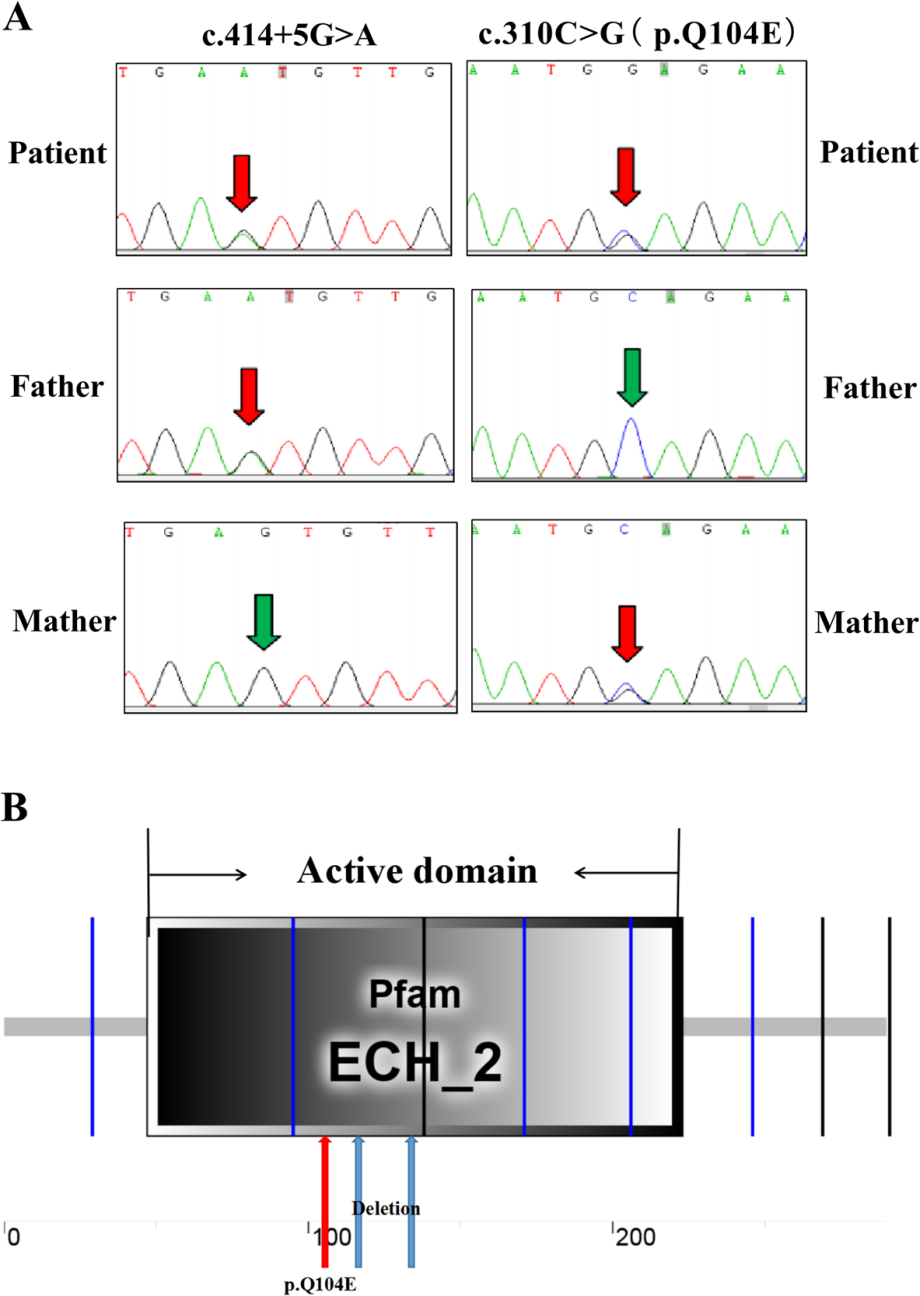
*ECHS1* Sanger sequence analysis and *ECHS1* functional domain. **a** Sanger sequencing of genomic DNA of patient and his parents. **b** A schematic diagram of the functional domains in ECHS1 and the locations of the mutation and deletion

Next generation sequencing was used to analyse the whole exome of the patient with suspected mitochondrial disorders. A noncoding rare heterozygous variant c.414 + 5G > A (chr10: g. 135,183,403) in intron 3 and a novel heterozygous variant c.310C > G (Chr10:135183512) in CDS of the *ECHS1* [MIM #602292] gene were found. No other changes were observed in the studied region. Because most mitochondrial diseases caused by nuclear DNA mutations are inherited in an autosomal recessive manner, the two novel variants seemed particularly peculiar. We proceeded to verify the genomic *ECHS1* DNA from both the patient and his parents using Sanger sequencing. The results revealed no other *ECHS1* variants. Furthermore, the patient’s father was heterozygous for only one variant, c.414 + 5G > A, and the mother for only the other variant, c.310C > G, indicating that the patient inherited one variant from each parent, and that both mutant alleles were expressed (Fig. 3). The information on the two novel variants is summarised in Table 1.

**Table 1:** The information of variants

Gene name	Chromosome localization (GRCh37/hg19)	Variant	NM	Location	Homozygosis/Heterozygosis	Amino acid alteration	Inheritance model	Variation source
*ECHS1*	Chr10:135183403	c.414 + 5G > A	NM_004092.3	Intron3	Heterozygosis	N/A	Autosomal recessive inheritance	Father
	Chr10:135183512	c.310C > G		CDS	Heterozygosis	p.Q104E	Autosomal recessive inheritance	Mather

We predicted the variant c.310C > G function by Phyre2 (http://www.sbg.bio.ic.ac.uk/phyre2/html/page.cgi?id=index) and I-TASSER (https://zhanglab.ccmb.med.umich.edu/I-TASSER/). The analysis indicated that this variant might change glutamine (Q) to glutamic acid (E) in the 104th amino acid sequence (p.Q104E).

**Fig. 4:**
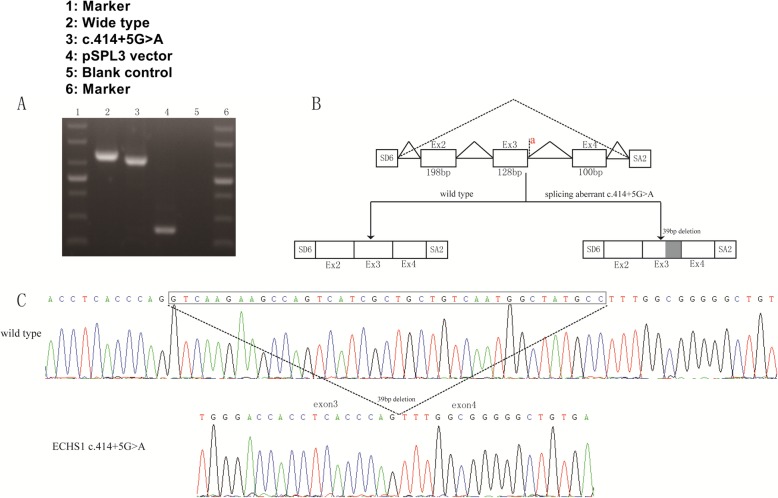
Minigene assay of c.414 + 5G > A variant. **a** The electrophoresis results of wild-type and c.414 + 5G > A transfections; **b** The schematic image of normal and aberrant splicing products; **c** Sanger sequence of wide type and c.414 + 5G > A variant

Because c.414 + 5G > A was located in an intron, we first performed bioinformatic analysis using the Human Splice Finder, Splice Port, and Fruit Fly Splice Predictor. The results showed high probability for intronic cryptic splice site activation, leading to 39 bp deletion. Splice site score calculator (http://rulai.cshl.edu/new_alt_exon_db2/HTML/score.html) was used to assess the strength of the constitutive and cryptic acceptor splicing sites, yielding 14.2 and 7.9, respectively. To prove that the 39 bp deletion was caused by the c.414 + 5G > A variant, we then conducted the minigene-based splicing experiment. The minigene splicing products were analysed by PCR amplification with plasmid-specific primers and visualised with polyacrylamide gel electrophoresis (Fig. 4a). The electrophoresis results of wild-type and c.414 + 5G > A transfections indicated that the c.414 + 5G > A variant exerted significant effects on splicing patterns. The amplicons confirmed by Sanger sequencing showed that the cDNA fragment obtained from the c.414 + 5G > A plasmid contained the same 39 bp deletion as the cDNA of the patient (Fig. 4c).

### In silico analysis of c.414 + 5G > A and c.310C > G variants in protein function

**Fig. 5:**
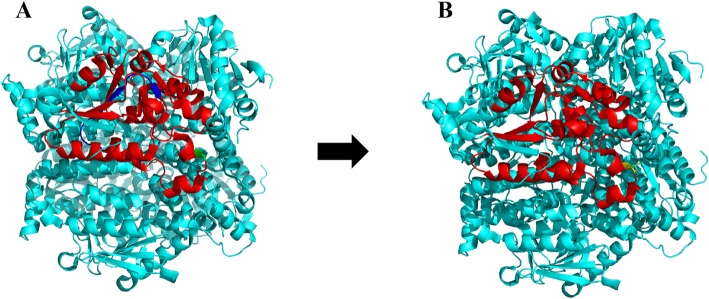
3D model of the human ECHS1. **a** Wide type, red-confidently predicted active domain, blue-deleted amino acids, green-glutamine (Q); **b** Mutated type, the number 126–138 amino acids deleted, yellow-glutamic acid (E)

To predict the impact of 39 bp deletion and p.Q104E caused by c.414 + 5G > A and c.310C > G on protein function, we performed in silico analysis. VariantValidator [5] was used to verify the annotation of the Chr10:135183403 deletion. The Human Splice Finder [6], Splice Port [7], and Fruit Fly Splice Predictor [8] were used to assess the predicted effect on the splicing site. SMART analysis (http://smart.embl-heidelberg.de) suggested that the catalytic domains of the core enzyme comprised amino acids 42 to 290 in the ECHS1 sequence. The 39 bp deletion in mature mRNA might cause a 13 amino acid-deletions (from amino acid 126 to 138) in the core enzyme catalytic region of ECHS1. The 13 missing amino acids and p.Q104E were both located in the protein active site. Subsequently, PyMol software was used to construct the 3D model of ECHS1 (Fig. 5). Such alteration with the involvement of highly conservative amino acids probably had a crucial impact on protein activity. Therefore, we speculated that the mutations severely reduced ECHS1 enzyme activity.

### Measurement of ECHS1 enzyme activity in patient’s myoblasts

**Fig. 6:**
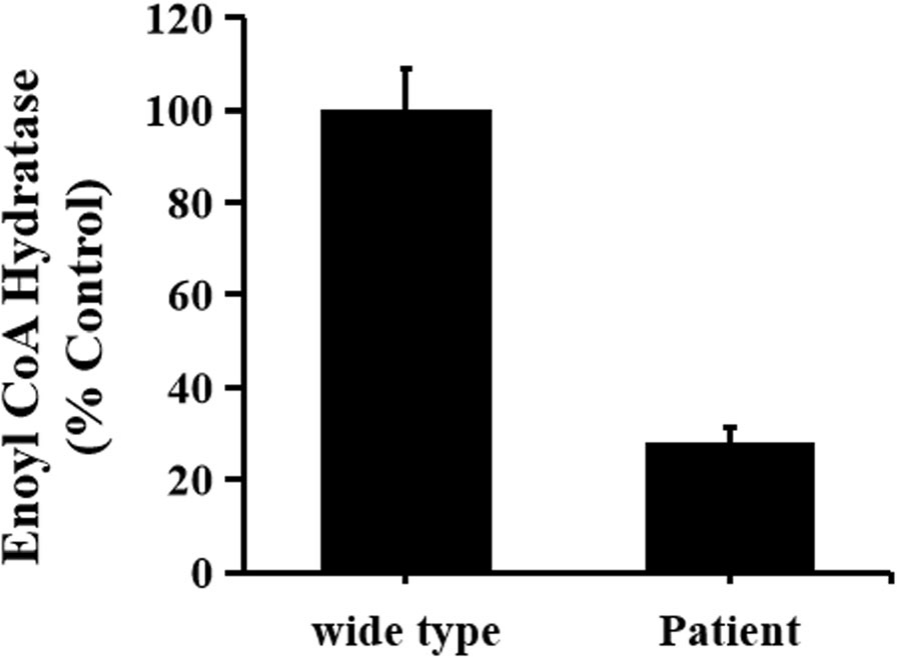
Mitochondrial fractions isolated from patient-derived myoblasts were used to estimate ECHS1 enzyme activity in the patient. The ECHS1 activity measurement was normalized to control sample

Based on the above computer prediction, in order to test the exact functional impact of the novel variants, we proceeded to determine if ECHS1 enzyme activity changed in the mitochondria of patient’ myoblasts. ECHS1 enzyme activity in the patient-derived primary fibroblast cell lysates was determined by spectrophotometry, followed by measuring the absorbance of the unsaturated substrate crotonyl-CoA over time (15 min). The experimental procedure was performed according to previous publications [9]. The experiments were performed in triplicates. Error bars represent standard deviations. Our results showed that ECHS1 enzyme activity in mitochondrial fractions of myoblast cell lysates from patients was remarkably reduced when normalised to control activity. ECHS1 enzyme activity from patients decreased to 28% of the normal value (Fig. 6). Overall, the variants might generate a serious depletion of ECHS1 enzyme activity.

## Discussion and conclusions

In this report, we identified a 2-year old patient harbouring two novel heterozygous variants, c.414 + 5G > A and c.310C > G, in *ECHS1*. To our knowledge, our study is the first to report the c.414 + 5G > A and c.310C > G variants, and provided the first experimental characterisation of the c.414 + 5G > A variant, elucidating its impact on splicing. The c.310C > G variant in the CDS region might result in a single amino acid mutation, changing glutamine (Q) into glutamic acid (E) in the number 104th amino acid sequences (p.Q104E). The c.414 + 5G > A variant was located in intron 3, and the variant might bring about 13 amino acid deletions. 3D structure prediction of ECHS1 showed that p.Q104E mutation and 13 amino acid deletions might destroy the activity domain. Surprisingly, we found that ECHS1 enzyme activity was lower in patient-derived myoblasts than in the control, which corroborates the previous speculation.

ECHS1 deficiency, as reported previously, is mostly characterised by the following prominent neurological symptoms: sensorineural deafness, developmental retardation, epileptic seizures, optic atrophy, and hypotonia. Other clinical features include cardiomyopathy, respiratory insufficiency, and elevated lactic acid levels in blood, urine, and cerebrospinal fluid. MRI images of the brain are similar to those of Leigh syndrome, with white matter changes, symmetrical high T2 signal in the bilateral basal ganglia, and brain atrophy [10, 11]. In our report, the patient presented with similar symptoms that were indicative of neurologic disorders, as well as elevated lactic acid levels. Regarding the association between *ECHS1* genetic variants and diseases, previous publications have reported different types of variants in patients. There were 34 pathogenic mutant forms, among which 29 were missense mutations, 3 were splicing mutations, 1 was a code shift mutation, and 1 was a nonsense mutation [1, 12]. Missense mutations and complex heterozygous mutations were found in the majority of the cases. Homozygous mutations, mostly found in exon 4, exon 5 and exon 6 as well as in exon 8, and splicing mutations, were also found in some cases. The most frequently studied variants were c.476A > G (p.Gln159Arg), c.538A > G (p.Thr180Ala), and c.817A > G (p.Lys273Glu) [1, 3, 13, 14]. Many types of variants were found; however, no clear association between the clinical phenotype and genotype of *ECHS1* was found. We presently found two novel variants, c.414 + 5G > A and c.310C > G, in the *ECHS1* gene, and no other variants were found in the study region. Both computer prediction and experimental analysis showed that ECHS1 enzyme activity was decreased in the patient’s myoblasts. However, there were some limitations to our study. First, we lacked an in vitro experiment, especially exogenous expression of the respective mutant ECHS1 protein in cancer cells, to verify the exact effects of these two variants on ECHS1 expression and enzyme activity. Second, a rescue assay is needed. Inserting wild-type ECHS1 into immortalised patient-derived myoblasts to observe the enzyme activity level and mitochondrial function, which requires several more patients, in order to determine the detailed association between these variants and disease.

In conclusion, two novel c.414 + 5G > A and c.310C > G variants leading to decreased ECHS1 activity were identified in our study. Establishing a diagnosis in patients with a proposed mitochondrial disorder is often a challenge, especially in paediatric cases. Therefore, we hope that these pathogenic variants could serve as biomarkers for mitochondrial encephalopathy, making the diagnosis of mitochondrial diseases simpler, more convenient, and more accurate.

## Data Availability

We declared that materials described in the manuscript, including all relevant raw data, will be freely available to any scientist wishing to use them for non-commercial purposes, without breaching participant confidentiality.

## Abbreviations

ECHS1: Enoyl-CoA hydratase
MRI: Magnetic Resonance Imaging
MRS: Magnetic Resonance Spectrum
EMG: Electromyography
